# The UV Plasmonic Behavior of Distorted Rhodium Nanocubes

**DOI:** 10.3390/nano7120425

**Published:** 2017-12-04

**Authors:** Yael Gutiérrez, Dolores Ortiz, José M. Saiz, Francisco González, Henry O. Everitt, Fernando Moreno

**Affiliations:** 1 Department of Applied Physics, University of Cantabria, Avda. Los Castros, s/n., 39005 Santander, Spain; gvelay@unican.es (Y.G.); ortizd@unican.es (D.O.); saizvj@unican.es (J.M.S.); gonzaleff@unican.es (F.G.); 2 Department of Physics, Duke University, Durham, NC 27708, USA; everitt@phy.duke.edu; 3 U.S. Army Aviation and Missile RD&E Center, Redstone Arsenal, Huntsville, AL 35898, USA

**Keywords:** UV plasmonics, rhodium, nanoparticles, photocatalysis

## Abstract

For applications of surface-enhanced spectroscopy and photocatalysis, the ultraviolet (UV) plasmonic behavior and charge distribution within rhodium nanocubes is explored by a detailed numerical analysis. The strongest plasmonic hot-spots and charge concentrations are located at the corners and edges of the nanocubes, exactly where they are the most spectroscopically and catalytically active. Because intense catalytic activity at corners and edges will reshape these nanoparticles, distortions of the cubical shape, including surface concavity, surface convexity, and rounded corners and edges, are also explored to quantify how significantly these distortions deteriorate their plasmonic and photocatalytic properties. The fact that the highest fields and highest carrier concentrations occur in the corners and edges of Rh nanocubes (NCs) confirms their tremendous potential for plasmon-enhanced spectroscopy and catalysis. It is shown that this opportunity is fortuitously enhanced by the fact that even higher field and charge concentrations reside at the interface between the metal nanoparticle and a dielectric or semiconductor support, precisely where the most chemically active sites are located.

## 1. Introduction

Nanoplasmonics explores and exploits when metallic nanoparticles are excited by electromagnetic radiation and the “free” electronic plasma of the metal oscillates at the incident frequency. Near $\omega_{p}/\sqrt{3}$, where $\omega_{p}$ is the plasma frequency, these oscillations reach their maximum amplitude, leading to a resonance known as Localized Surface Plasmon Resonance (LSPR). This produces two important effects: a localization of the electromagnetic radiation at a scale much smaller than the incident wavelength, and a local enhancement of the electromagnetic field proximal to the nanoparticle [1,2,3]. Traditionally, nanoplasmonics have studied noble metals Au, Ag, Cu or their composites [4,5,6] whose LSPRs are in the visible (Vis) or near-infrared (NIR) spectral regions. Recent interest in ultraviolet (UV) nanoplasmonics offers new opportunities in surface-enhanced Raman spectroscopy (SERS) [7,8,9], photocatalysis [10,11,12,13,14], and biology [15,16]. Aluminum is a particularly promising metal for UV nanoplasmonics because it is one of the most abundant materials in the Earth’s crust, it is inexpensive, and it has compelling electromagnetic properties [17,18,19,20] . However, Al suffers from the formation of an oxide layer several nanometers thick that limits its UV plasmonic performance and utility for applications such as plasmon-assisted photocatalysis requiring contact with the metal surface [18,21,22]. Magnesium, another promising metal for UV plasmonics, forms an even more aggressive oxide, which severely limits its utility [19,22,23,24]. Although Gallium is an appealing metal for UV plasmonics because of its self terminating oxide layer being only a monolayer thick [25,26,27], it has lower electrical conductivity and presents a liquid–solid phase transition near room temperature that hinders its manipulation.

A particularly intriguing metal whose UV plasmonic behavior has only recently been discovered is rhodium, already well known for its catalytic activity produced by a partially filled d-shell and its commensurately low tendency to oxidize [9,19,28,29,30,31]. Rh nanoparticles (NPs) may be synthesized into a variety of shapes through chemical methods with nanometer size control [9,29,30]. Already, size-controlled Rh tripod stars and nanocubes (NCs) have been grown, a systematic numerical study has been performed for the tripod stars, and activity for UV SERS, surface enhanced fluorescence, and photoinduced degradation of p-aminothiophenol has been demonstrated [9,28,29]. However, its most exciting potential may lie with the possibility of enhancing rhodium’s already favorable catalytic activity by UV illumination near its plasmonic resonance. Very recently, Zhang et al. discovered that Rh NCs on Al${}_{2}$O${}_{3}$ supports exhibit plasmonic photocatalytic activity in the carbon dioxide methanation reaction by simultaneously lowering the activation energy of the rate determining step and strongly selecting the desired product CH${}_{4}$ over the undesired product CO [30].

In plasmonic photocatalysis, chemical reactions are driven on the surface of illuminated metallic NPs [32,33]. The most commonly accepted mechanism for this is the so called direct transfer mechanism, by which excitation of the LSPR generates hot carriers that migrate to the surface and enter anti-bonding orbitals of adsorbed molecules, thereby weakening a critical bond and accelerating the reaction. Since the LSPR of the NP drives this mechanism, mapping the location of the surface electromagnetic and carrier hot-spots will indicate regions in which the photochemical processes are enhanced. For thermal catalysis, it is well known that the most active sites on metal NPs are corners and edges because adsorbed chemical intermediates may have higher coordination with surface atoms there [31,34]. Therefore, for plasmonic photocatalysis to be effective, the photogenerated hot carriers from LSPR decay must also reach these most active sites.

Here, we report a detailed numerical analysis of the UV plasmonic behavior and charge distribution of Rh NCs, a geometry easily fabricated with precise size control [29]. It will be shown that the strongest plasmonic hot-spots are located at the corners and edges of the NCs, exactly where they are the most catalytically active, and that distortions of the cubical shape significantly affect their plasmonic and photocatalytic properties. The fact that the highest fields and highest carrier concentrations occur in the corners and edges of Rh NCs confirms their tremendous potential for plasmon-enhanced catalysis. Transmission electron microscopy of the largest Rh NCs indicated that the surfaces are not flat but slightly concave with even more pointed corners, so the effects of surface concavity will be explored to understand how the lightning rod effect concentrates field and carriers there. Because intense catalytic activity at corners and edges will reshape these regions, we also explore how the electromagnetic and charge distributions evolve as the shape evolves from concave cubical to convex cubical to spherical with corners and edges of increasing radii of curvature. By this, we can quantify how the photocatalytic activity of these nanostructures deteriorates, a critical concern for the practical application of any catalyst. Finally, we explore how the charge distribution is affected when these NCs are on a dielectric (Al${}_{2}$O${}_{3}$) support, where again it is fortuitously demonstrated that the most chemically active sites at the interface of the metal NC and the support are where the highest field and charge concentrations reside.

## 2. Theoretical Methods

The electromagnetic interaction between the Rh NCs and light has been modeled using finite element (FEM) simulations implemented using the commercial software COMSOL Multiphysics 5.2 (COMSOL Inc., Burlington, MA, USA) [35]. Its radio frequency (RF) module allows us to formulate and solve the differential form of Maxwell’s equations together with initial and boundary conditions. The equations are solved using the finite element method with numerically stable edge element discretization in combination with state-of-the-art algorithms for preconditioning and solving the resulting sparse systems of equations.

A spherical region of embedding medium, whose radius is larger than the illuminating wavelength, is modeled around the NP. A perfectly match layer (PML) that acts as an absorber of the scattered field is placed outside of the embedding medium domain. The mesh was fine enough as to allow convergence of the results. Its maximum element size inside the NP was set around a tenth of the minimum skin depth over the spectral range.

The incident power density *S* is defined as

(1) $$S=\frac{|\vec{E}{\left(r,t\right)|}^{2}}{Z_{sm}},$$

where $\vec{E}\left(r,t\right)$ is the local electric field, and $Z_{sm}$ is the impedance of the embedding medium, calculated by

(2) $$Z_{sm}=\frac{Z_{0}}{n_{sm}}.$$

Here, $Z_{0}=\mu_{0}c$ is the impedance of free space (with $\mu_{0}$ being the vacuum permeability and *c* the speed of light in free space), and $n_{sm}$ is the refractive index of the embedding medium. The absorption cross-section $C_{abs}$ can be calculated as the integral of the resistive losses over the NP’s volume, normalized to the incident power density *S*. The surface charge density σ can be calculated according to the conservation of normal component of the displacement vector $\vec{D}$ across the metal-embedding medium boundary.

The spectral dependence of three parameters has been studied: the absorption cross-section ($C_{abs}$), the local field enhancement of the electric field (${\left|E\right|}^{2}$), and the surface charge density (σ), all of which depend on the energy of the illuminating electromagnetic field. For particles much smaller than the wavelength, $C_{abs}$ can be associated with the far-field extinction efficiency and helps to describe the ellipsometric response of surfaces with metallic nanostructures, especially those related to the imaginary part of the measured pseudodielectric function [36]. ${\left|E\right|}^{2}$ represents the local field enhancement. The areas with greatly enhanced field intensity, the so called hot-spots, are the regions where the hot-electron generation process takes place. The evaluation of ${\left|E\right|}^{2}$ allows us to identify the regions over the NP surface where hot electrons are being generated [37], this information being useful for photocatalysis research [32]. A deeper analysis of this aspect is made through the evaluation of σ: the regions with highest charge concentrations are the most reactive with the highest probability of charge transfer.

**Figure 1:**
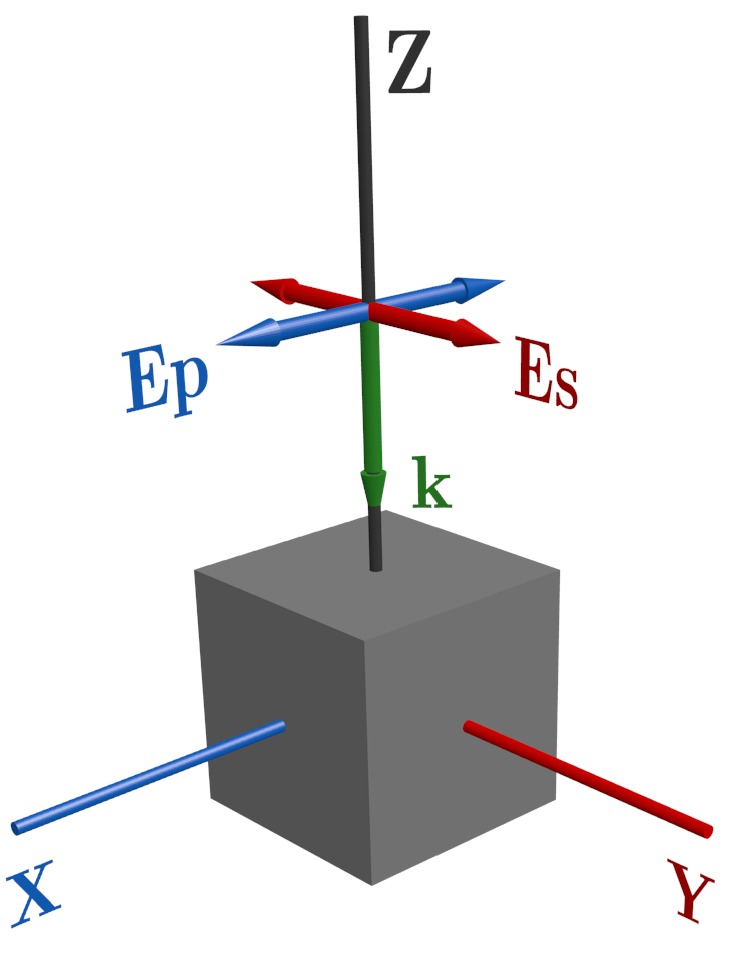
Diagram of a nanocube. It is illuminated by a monochromatic plane wave propagating ($\vec{k}$) along the *z*-axis with either P- or S-polarization.

We have performed finite element simulations on Rh nanostructures and have compared them with identical ones made of Ag. These NPs have been illuminated with a monochromatic linearly polarized plane wave within a spectral interval of interest: the illumination geometry (wave vectors and polarization) is properly described in Figure 1.

**Figure 2:**
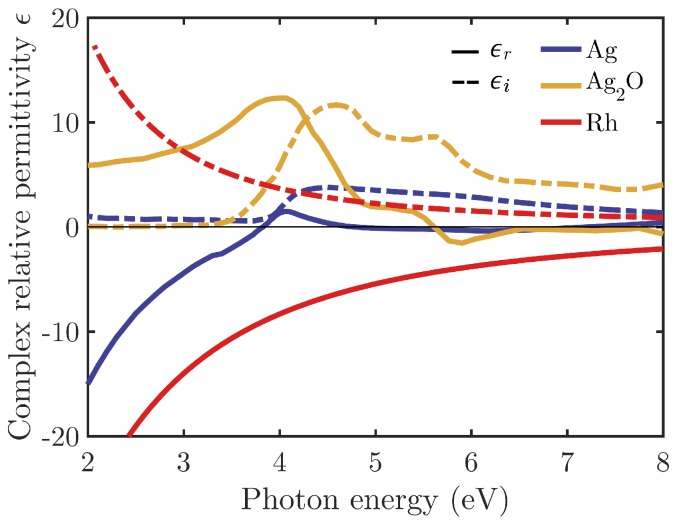
Real (solid line) and imaginary (dashed line) parts of the complex relative permittivity (ϵ) of Ag (blue), Ag${}_{2}$O (yellow) and Rh (red) as a function of the photon energy of the incident beam.

In this work, we use the complex dielectric functions of Rh and Ag shown in Figure 2. The oxide Ag${}_{2}$O that constitutes the Ag coating is also included. These values have been obtained from different sources in the literature [38,39]. Figure 2 shows the complex dielectric permittivity of these materials. The Frölich frequency ($\epsilon_{r}=-2$) of Ag lies in the near UV, but Rhs are deep in the UV region. Furthermore, Ag presents interband transitions in the UV that inhibit plasmonic performance in this spectral range.

## 3. Results and Discussion

### 3.1. Comparison of Ag and Rh in Simple Geometries

Silver, when synthesized in cubic NPs, has been shown to present good plasmonic performance and has been used as a metallic photocatalytic enhancer below 3 eV [32]. Oxidation and strong interband transitions above 3 eV prevent its use for applications in the UV range, especially when the size is below 10 nm, as preferred in photocalysis. In this section, we present a direct comparison of cubical silver and rhodium NPs to show how the latter presents two clear advantages for UV applications: its lack of oxidation, especially important for NPs of very small sizes (less than 10 nm [9,29]), and its lack of interband transitions above 3 eV.

As a first step, we consider cubic monomers of Rh and Ag with an edge length of 30 nm and rounded edges (curvature radius $R_{c}=2$ nm). This size has been chosen because it allows a good comparison with silver when oxidation effects have no appreciable effect, and also because this size has been recently used in photocatalysis and SERS experiments with rhodium [30]. For smaller sizes [9,29], silver becomes useless because oxidation destroys its plasmonic performance. The nanostructure is illuminated with a monochromatic plane wave under normal incidence and P-polarization (see Figure 1). To explore the relative contributions of the dielectric function and the geometry, calculations for a sphere and a disk with equivalent volume are also presented. The radius of the equivalent sphere is R=18.6 nm. The equivalent disk is considered to have the same radius *R* and height *h* (R=h=20.5 nm).

**Figure 3:**
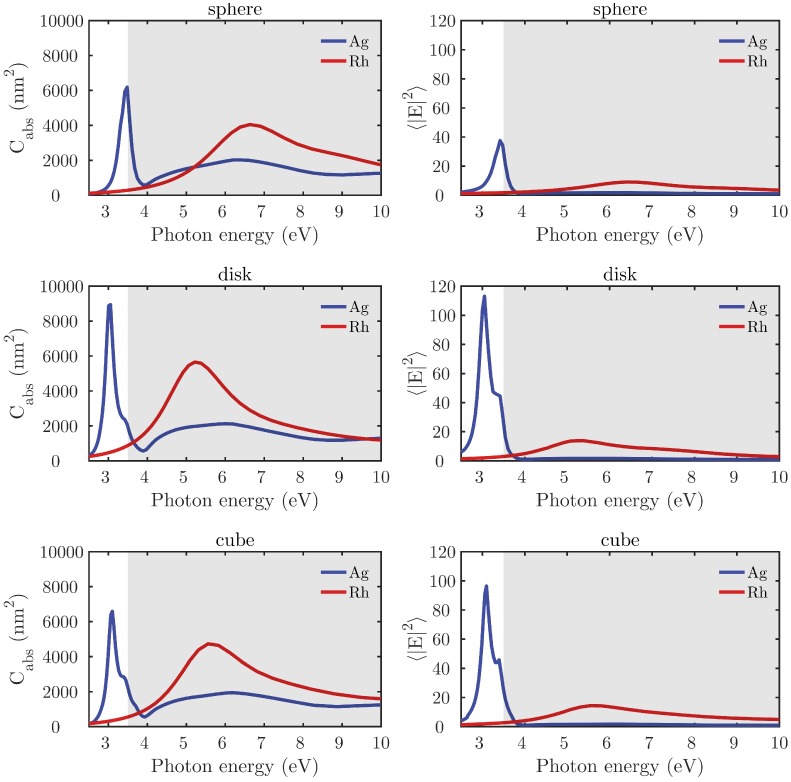
Absorption cross-section ($C_{abs}$) (**left column**) and spectral electric field intensity ($\langle |E{|}^{2}\rangle$) averaged over the nanoparticle’s closed surface (**right column**) for spheres (R=18.6 nm), disks (R=h=20.5 nm), and cubes (L=30 nm) composed of Rh (red) or Ag (blue) illuminated under normal incidence, and embedded either in air.

Figure 3 plots the absorption cross-section $C_{abs}$ (left column) and spectral electric field intensity averaged ($\langle |E{|}^{2}\rangle$) over the NP’s closed surface (right column) for equivalent spheres, disks, and cubes made of Rh and Ag. As expected, the two metals have different behaviors: Ag presents LSPRs within the interval 3–4 eV, whereas the Rh plasmonic response is located between 4 and 10 eV. This behavior is commensurate with their respective values of the Fröhlich energies $E_{f}$ [19] (E${}_{f}^{Ag}$ = 3.5 eV and E${}_{f}^{Rh}$ = 8.2 eV ). In addition, it can be observed how the shape of NP has a strong influence on the LPSRs and their spectral position.

### 3.2. Rh Nanocubes

#### 3.2.1. Effect of the Shape

Given this strong dependence of the optical response on the geometry of the NP [40], we now consider the dependence of the LSPR for an isolated NC with the deviations from the perfect cubical geometry that could appear during chemical synthesis or from the extended use of the NCs as catalysts. Specifically, we will study the effect of rounding the edges and corners of the NC with different radius of curvature ($R_{c}$) varying from 0 (perfect cube) to 15 nm (perfect sphere) while keeping the distance between flat opposite faces constant. The effect of introducing a concavity (decreasing volume) or convexity (increasing volume) from 0 (flat) to 4 nm (most depressed or extended) on the faces of the NC will be also analyzed. Both studies have been done starting from a perfect NC with side length L=30 nm illuminated with a monochromatic plane wave under normal incidence and P-polarization.

**Figure 4:**
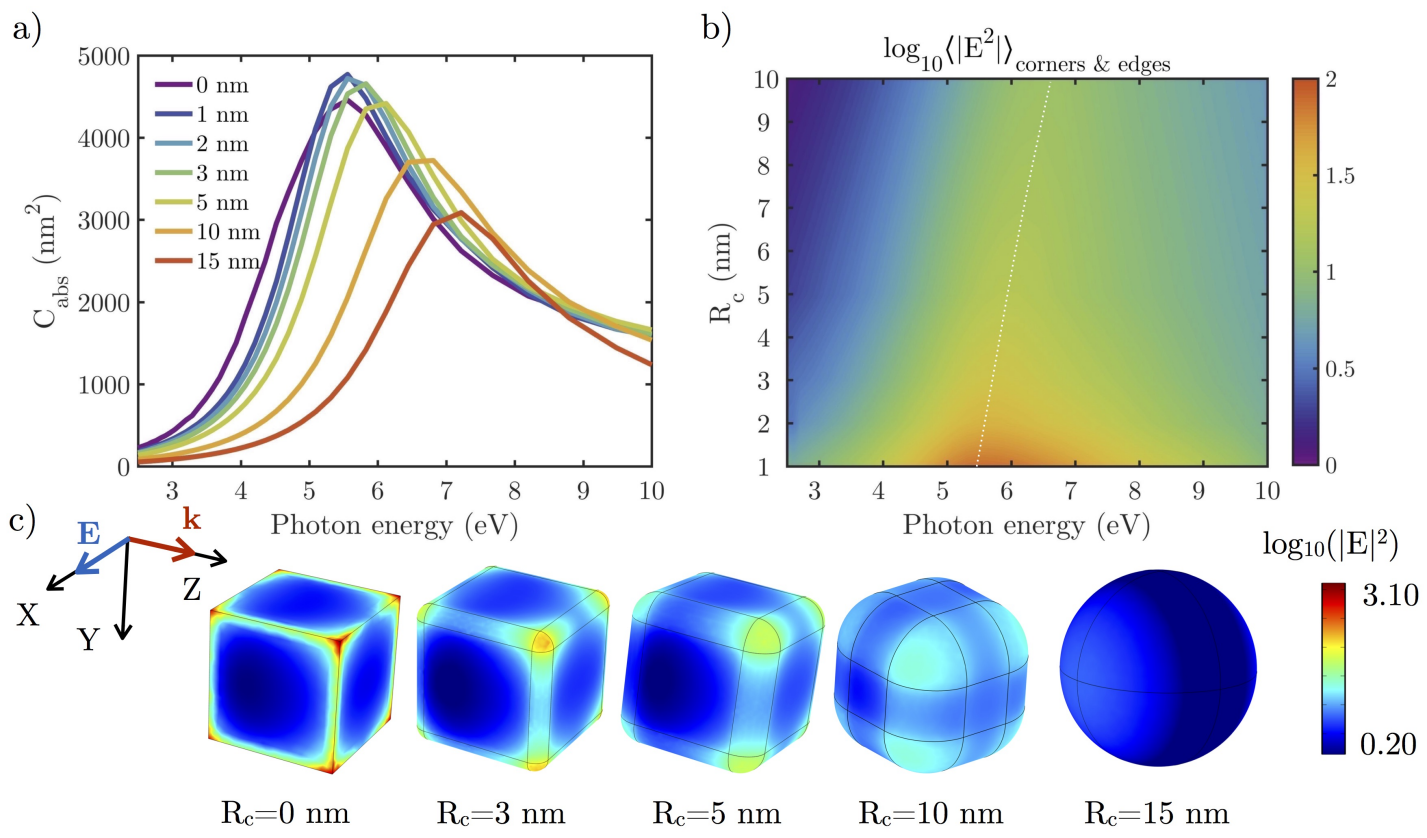
(**a**) absorption cross-sections ($C_{abs}$) of edge length L=30 nm Rh NCs with rounded edges and corners with a curvature radii $R_{c}$ varying from 0 (perfect cube) to 15 nm (perfect sphere); (**b**) local field enhancement (${\left|E\right|}^{2}$) averaged over the corners and edges (where charge is most concentrated) of the NC with different $R_{c}$; (**c**) local field enhancement in logarithmic scale (${\left|E\right|}^{2}$) over the surface of the different NCs when illuminated with the corresponding resonant wavelength.

Figure 4 shows the effect on the absorption cross-section $C_{abs}$ and the local field enhancement ${\left|E\right|}^{2}$ of rounding the edges of an NC with L=30 nm.

When increasing $R_{c}$, two main effects can be seen in the absorption cross-section spectra: a blue-shift and a decrease of the resonance peak caused by competing effects. The first is associated with the volume reduction as NCs evolve from a perfect cube to a perfect sphere (27,000 to 14,137 nm${}^{3}$) while keeping constant the distance between the center of opposite faces. In addition, the amplitude of charge oscillations is reduced because of less localized charges when the NC edges and corners are rounded. By looking at the near-field (NF) distribution over the particle surface (Figure 4b,c), it can be seen how the hot-spots are less intense and charge density less localized as $R_{c}$ increases. In these cases, the depolarizing field, which appears as reaction to the applied field [41,42] is weaker, leading to resonance peaks at higher energies. In these cases, it is important to emphasize that the near-field resonance (Figure 4b) is red-shifted in comparison to the far-field one ($C_{abs}$), as has been pointed out previously [43,44]. For rhodium, this shift is small (≈0.1 eV for a plasmon resonance located on 6 eV), so far-field magnitudes like $C_{abs}$ give accurate enough spectral information about what is happening close to the particle.

**Figure 5:**
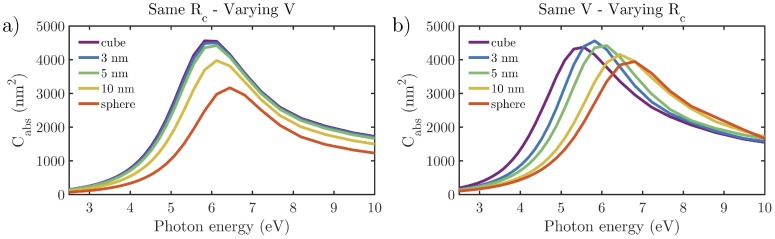
Absorption cross-sections ($C_{abs}$) spectra of (**a**) cubes with $R_{c}=5$ nm with the same volume as those with L=30 nm and $R_{c}$ indicated by the legend and (**b**) cubes with the same volume as a cube with L=30 nm and $R_{c}=5$ nm, but with an $R_{c}$ given by the legend.

In order to discriminate the contribution to $C_{abs}$ of both aforementioned aspects separately, we consider a reference NC with L=30 nm and $R_{c}=5$ nm. Figure 5a shows the effect of varying the volume of the nanocube while keeping $R_{c}=5$ nm constant, and Figure 5b illustrates the effect of varying $R_{c}$ while keeping the volume of the NP constant. The spectral blue-shift introduced by these effects are ΔE=0.4 eV and ΔE=1.2 eV, respectively caused by volume reduction and charge delocalization. This blue-shift is also manifested in Figure 4b, where the spectral values of ${\left|E\right|}^{2}$ averaged over the rounded edges and corners of the NCs decreases an order of magnitude as $R_{c}$ is reduced from 1 to 10 nm.

**Figure 6:**
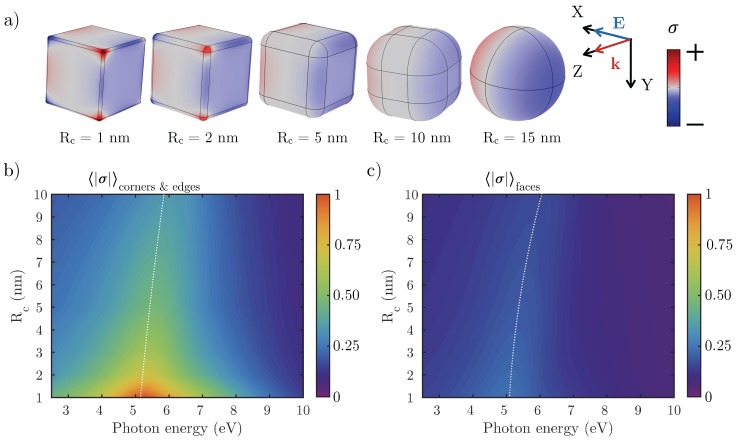
(**a**) surface charge distribution (σ) over Rh NCs with rounded edges and tips with a curvature radii $R_{c}$ when illuminated at the resonant wavelength. The red/blue regions represent positive/negative charge densities; (**b**) normalized values of σ averaged over the edges and corners; and (**c**) over the flat faces of the NCs.

To better illustrate the aforementioned delocalization of the charge distribution, Figure 6a shows the surface charge density (σ) on the cube’s faces as $R_{c}$ increases. Large values of σ are associated with a high chemical reactivity [31,34]. The red/blue regions represent positive/negative charge densities. Figure 6b,c show the normalized absolute value of the averaged surface charge density 〈|σ|〉 on the corners and edges, and on the flat faces of the cube, respectively. It can be seen how the highest concentrations of surface charge density are produced at the corners and edges of NCs with smallest $R_{c}$. As we go from $R_{c}=1$ to $R_{c}=4$ nm, the value of 〈|σ|〉 goes down to 1/4 of its original value. However, the value 〈|σ|〉 on the faces remains almost constant no matter the value of $R_{c}$.

Experimentally, it has been observed that, as the cubes are re-used in photocatalytic processes, they are reshaped. The longer the catalyst is used, the more spherical they become, leading to a loss of reactivity. This effect is related with the lowering of σ as $R_{c}$ increases [31].

**Figure 7:**
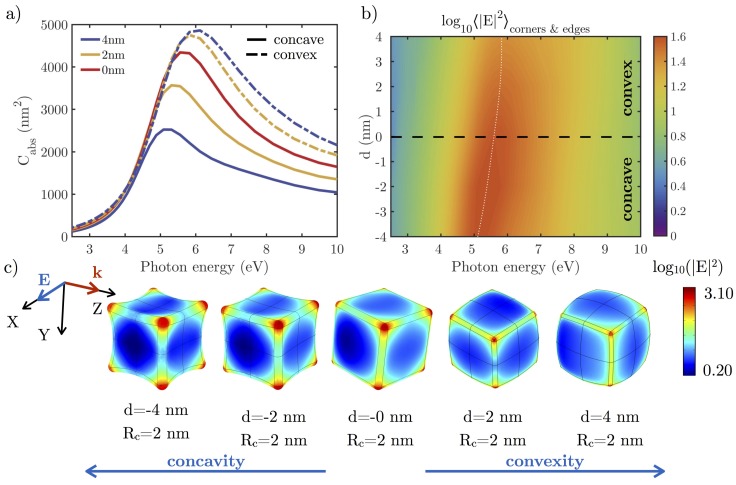
(**a**) absorption cross-sections ($C_{abs}$) of L=30 nm and $R_{c}=2$ nm Rh NCs as a function of concave/convex deformation parameter *d* indicated in the legend; (**b**) local field enhancement (${\left|E\right|}^{2}$) averaged over the corners and edges of NC with different *d*; (**c**) local field enhancement in logarithmic scale (${\left|E\right|}^{2}$) over the surface of the different NCs when illuminated at resonance.

Figure 7 shows the effect on the absorption cross-section $C_{abs}$ and the local field enhancement ${\left|E\right|}^{2}$ when a concavity/convexity is introduced in an NC with L=30 nm and $R_{c}=2$ nm. This deformation is parametrized by *d*, which indicates the maximum amount that the convex or concave surface is deformed above or below the flat surface of the original NC. Positive/negative values of *d* are associated with a convex/concave deformation.

As convexity and volume increases, a blue-shift of $C_{abs}$ is produced, while $C_{abs}$ red-shifts when a concavity increases and volume decreases. This seems to be in contradiction with preview observation that increasing/decreasing the volume of an NP produces a red/blue-shift of the resonance peak due to an increase/decrease of the depolarizing field inside the particle [41]. However, as in the previous case, it is necessary to consider how the charge is distributed in each case. The lightning rod effect [45] states that the charge density is higher in sharper tips. If we compare both concave and convex NCs, it can be seen how the concave NCs have sharper corners and edges, so the charge density is higher in these regions, leading to a more intense depolarizing field that causes the resonance peak to red-shift. The great sensitivity of plasmonic response to the degree of deformation agrees with experimental results obtained by Romo-Herrera et al. in gold NCs [46].

Figure 7b shows the local field enhancement ${\left|E\right|}^{2}$ averaged over the corners and edges of the nanocubes. It can be seen that the maximum value also blue-shifts as NCs go from concave to convex. In addition, the maximum value ${\left|E\right|}^{2}$ decreases as *d* is increased. The distribution of ${\left|E\right|}^{2}$ over the particle surface is shown in Figure 7c. Not only do concave cubes present higher values of ${\left|E\right|}^{2}$ in the corners and edges, they also represent a larger fraction of the surface. Concave cubes are therefore expected to be more reactive than convex ones.

**Figure 8:**
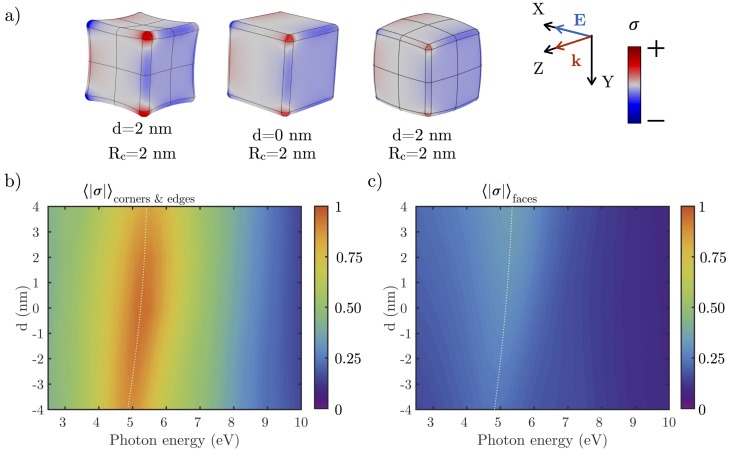
(**a**) surface charge distribution (σ) over concave and convex Rh NCs with L=30 nm and $R_{c}=2$ nm when illuminated at resonance. The red/blue regions represent positive/negative charge densities. Normalized values of σ averaged over (**b**) the edges and corners; and (**c**) over their deformed faces of the NCs.

Figure 8a shows the surface charge density σ over an Rh NC with $R_{c}=2$ nm and d=2 nm concave/convex deformation when illuminated at resonance. Higher concentrations of charge can be found on the edges and corners of the NC. Figure 8 shows the normalized absolute value of the averaged surface charge density 〈|σ|〉 over (b) corners and edges, and (c) the deformed faces of the cube. The values of 〈|σ|〉 on the corners and edges are ≈2.5 times greater than on the faces. Higher values of *d* lower 〈|σ|〉. The value of 〈|σ|〉 on the faces remains almost constant, increasing slightly as the cube becomes more convex.

#### 3.2.2. Dielectric Substrate

**Figure 9:**
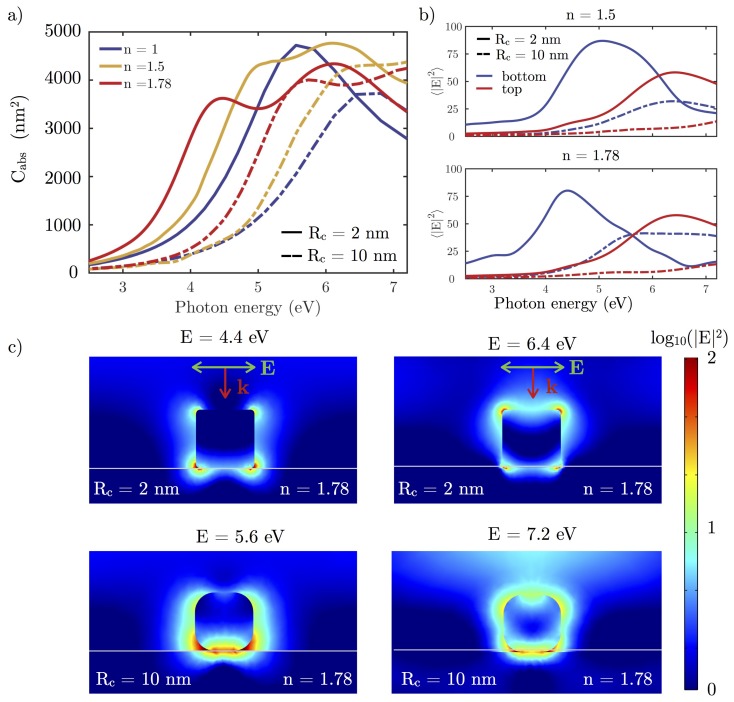
(**a**) absorption cross-section ($C_{abs}$) of a L=30 nm and $R_{c}=2/10$ nm (solid/dashed line) Rh NC under normal incidence with a P-polarization beam on substrates with different refractive indexes; (**b**) ${\left|E\right|}^{2}$ averaged over the corners and edges in the bottom (contact with the substrate, blue line) and top (red line) faces of the cube on a glass (n=1.5) and sapphire (n=1.78) substrates; (**c**) logarithmic scale ${\left|E\right|}^{2}$ near-field maps for $R_{c}=2/10$ nm Rh NC (top/bottom) on sapphire substrate (n=1.78) at E=4.4/5.6 eV and E=6.4/7.2 eV.

To mimic common experimental configurations in SERS and photocatalysis [26,36,47], we consider an Rh NC located on a dielectric substrate for different refractive indeces. Examples commonly used in experiments are glass or sapphire with refractive indices of 1.5 and 1.78, respectively. Figure 9a plots the absorption cross-section ($C_{abs}$) for L=30 nm Rh/Ag NCs with two $R_{c}$ (2 and 10 nm) on different dielectric substrates. As the refractive index of the substrate increases, two peaks in the $C_{abs}$ spectra appear, a phenomenon previously reported by Sherry et al. [48] on Ag NCs. One of these two peaks is slightly blue-shifted with respect to the peak of the isolated cube, and the other is red-shifted. The latter is associated with large fields near the surface of the substrate, whereas the former is associated with large fields away from the substrate. This shift towards lower energies can be understood by considering that the corners and edges of the cube touching the substrate are surrounded by a higher effective refractive index [19,36,47] (see Figure 9c). These results are in agreement with those reported by Nicoletti et al. [49], who studied Ag NCs on a dielectric substrate through electron energy-loss spectroscopy (EELS).

Figure 9b shows the value of ${\left|E\right|}^{2}$ averaged over the edges and corners of the bottom (in contact with the substrate, blue lines) and top faces (in contact with air, red lines) of the Rh NC. The peak associated with the bottom face is red-shifted with respect to the top face peak because the corners and edges of the bottom face are surrounded by a higher refractive index than the top ones. In fact, the peak associated with the sapphire substrate is shifted towards lower energies than the one corresponding to the glass because the refractive index of sapphire is greater than glass (1.78 vs. 1.5). Conversely, the spectral value of ${\left|E\right|}^{2}$ averaged over the edges and corners of the top face (in contact with air) take approximately the same values no matter the substrate. The maximum average enhancement at the edges in contact with the substrate is higher than for those at the top face. This means that, at the interface between particle and substrate, a large concentration of plasmonically-excited carriers is produced, making this region the most reactive location.

Two ${\left|E\right|}^{2}$ near-field maps for cubes with $R_{c}=2$ and 10 nm on a sapphire substrate are plotted in Figure 9c, respectively. The chosen photon energies for each map are those at which the aforementioned averages take their maximum value: E=4.4/5.6 eV in the case of the bottom face, and E=6.4/7.2 eV for the top face. It can be seen how, on the bottom surface, very intense hot spots are produced in the interface between the cube an the substrate. However, there are still hot spots on the top, especially at the top corners of the NC. The main differences between the results for each value of $R_{c}$ analyzed are that for $R_{c}=10$ nm Rh NC, the spectra are blue-shifted with respect to those of $R_{c}=2$ nm, in accordance with the results reported in Figure 4a for isolated NCs. In addition, the values ${\left|E\right|}^{2}$ are lower for the $R_{c}=10$ nm Rh NCs as for the case of the isolated NCs (see Figure 4b,c).

**Figure 10:**
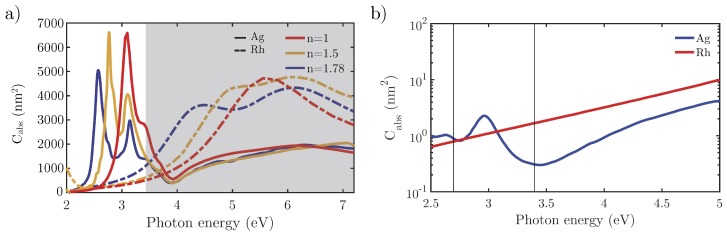
(**a**) absorption cross-section ($C_{abs}$) of a L=30 nm and $R_{c}=2$ nm Rh (dashed line) and Ag (solid line) NC under normal incidence with a P-polarization beam on substrates with different refractive indices. The shadowed region corresponds to the UV range; (**b**) absorption cross-section ($C_{abs}$) for L=5 nm Rh and Ag (with 1 nm Ag${}_{2}$O shell). The vertical lines indicate two commercial wavelengths ( 460 nm (2.7 eV) and 365 nm (3.4 eV), respectively).

Figure 10a compares the $C_{abs}$ spectra for Ag and Rh L=30 nm and $R_{c}=2$ nm NCs on glass and sapphire substrates. The spectra for isolated NCs are plotted too (red lines). As mentioned previously, Ag NCs are restricted for applications below the 3.5 eV because of its interband transitions, while Rh can operate in a wide range of the UV (4–7 eV).

As a final remark, consider that some commercial low cost light sources used in photocatalysis experiments operate in the near-UV and blue regions of the spectrum [30]. Optimum photocatalysis requires a good catalyst metal with efficient plasmonic behavior in these spectral ranges. Ag shows an excellent plasmonic response around 3 eV, but it suffers from oxidation and is a poor catalyst. Although Rh has a weaker plasmonic response in the near-UV, it is much better than other catalytic metals such as Pt or Pd [19], and its catalytic behavior is very good even off resonance. Figure 10b shows the absorption efficiency of a 5 nm Rh NC as compared to that of a Ag NC of the same size with 1 nm Ag${}_{2}$O shell. These sizes have been chosen because they are typical in photocatalysis experiments [50,51,52]. As can be observed, above 3 eV, and in particular for widely available UV sources at 365 nm (3.4 eV) marked with a vertical line in the plot, Rh has better plasmonic and photocatalytic performance than Ag.

## 4. Conclusions

This research is a consequence of the recent interest in UV plasmonics, the consequent search for new metallic materials with good UV plasmonic performance, and enhancement of chemical reactions assisted by light such as photosynthesis and photocatalysis. Because rhodium has good plasmonic and catalytic behaviors, we have numerically studied the electromagnetic behavior of NPs made of Rh with cubical geometry in the UV range. This study has been performed by analyzing practical parameters like their absorption cross-section, local field enhancement, and surface charge density. From this study, the general conclusion is that Rh is a promising candidate for applications in plasmon enhanced spectroscopy and catalysis. Through chemical synthesis and repeated photocatalytic processes, NCs may have concave/convex faces or rounded corners and edges. In this research, we have analyzed their effect on the plasmonic response of Rh nanocubes. In general, a concavity/convexity on the NC faces leads to a blue/red-shift of the LSPR peak. Depositing the cube on a substrate or rounding its edges and corners also generates a red/blue-shift of the resonance peak. Both the local field enhancement and the surface charge density reach their highest values at both the edges and the corners of the NC. The smaller the curvature radius of the edges and corners, the higher the values of the local field enhancement and surface charge density. These results serve as a guide to experimentalists for how Rh NC resonances may be optimized for the photocatalytic process or SERS-like applications, leading to a control of the near-field and surface charge densities over the NP surface.
